# Impacts of dietary calcium, phytate, and nonphytate phosphorus concentrations in the presence or absence of phytase on inositol hexakisphosphate (IP6) degradation in different segments of broilers digestive tract

**DOI:** 10.3382/ps/pev354

**Published:** 2016-01-06

**Authors:** W. Li, R. Angel, S.-W. Kim, K. Brady, S. Yu, P. W. Plumstead

**Affiliations:** * Department of Animal and Avian Sciences, University of Maryland, College Park, MD; † Enzyme R&D, DuPont Industrial Biosciences, Aarhus, Denmark; ‡ Danisco Animal Nutrition, DuPont Industrial Biosciences, Marlborough, UK

**Keywords:** IP6 concentration, crop, proventriculus and gizzard, ileum, phytase

## Abstract

A total of 1,440 straight-run Heritage 56M × fast-feathering Cobb 500F broiler birds were fed from 11 to 13 d of age to determine the impacts of calcium (**Ca**), phytate phosphorus (**PP**), nonphytate P (**nPP**) and phytase concentrations on the *myo*-inositol hexakisphosphate (**IP6**) flow through the different parts of gastrointestinal tract (**GIT**). The experiment was a 2×2×2×3 randomized block design with 2 Ca (0.7 and 1.0%), 2 PP (0.23 and 0.34%), 2 nPP (0.28 and 0.45%) and 3 phytase (0-, 500-, and 1,000-phytase unit (**FTU**)/kg) concentrations. The experiment was replicated twice (block) with 3 replicates per treatment (**Trt**) of 10 birds per block. Concentration of IP6 in crop, proventriculus (**Prov**) plus **(+)** gizzard (**Giz**) and distal ileum digesta as well as the ileal IP6 disappearance was determined at 13 d of age. In crop, higher IP6 concentration was seen with increased Ca (*P* < 0.05). Despite the interaction between PP and phytase, higher dietary PP led to greater IP6 concentration (*P* < 0.05). Similar main effects of PP and phytase were also seen in Prov+Giz and ileum (*P* < 0.05) without interactions. Interaction between Ca and nPP on IP6 concentration was seen in Prov+Giz (*P* < 0.05). Decreased ileal IP6 disappearance was found at higher Ca (62.3% at 0.7% Ca vs. 57.5% at 1.0% Ca; *P* < 0.05). In general, adding phytase improved IP6 degradation but the degree of impact was dependent on nPP and PP (*P* < 0.05). In conclusion, phytase inclusion significantly reduced IP6 concentration and IP6 disappearance in distal ileum regardless of GIT segments or diet composition, but impacts of dietary Ca, nPP, and PP differed depending on GIT segment examined.

## INTRODUCTION

Phytate (*myo*-inositol hexakisphosphate, **IP6**), is the principal phosphorus (**P**) storage form in plant seeds (Kemme et al., 1999). In typical corn and soybean meal (**SBM**) based poultry diets, between 40 to 60% of total P is presented as phytate P (**PP**) (NRC, 1994; NRC, 2013). The determined bioavailability of PP, varies from 0 (Nelson, 1976) to over 60% (Mohammed et al., 1991; Tamim et al., 2004) in poultry species and is affected by dietary factors, especially calcium (**Ca**) (Tamim and Angel, 2003; Tamim et al., 2004; Amerah et al., 2014), birds’ age (Li et al., 2014) and adaptation to P deficiencies (Li et al., 2014).

In poultry species, improved P utilization due to microbial phytase inclusion has been reported and reviewed extensively during the past two decades (Angel et al., 2002; Selle and Ravindran, 2007; Singh 2008; Selle et al., 2009; Adeola and Cowieson, 2011). Among the majority of the current commercial phytases, pH optima for maximum activity is between 3 and 5 (Brejnholt et al., 2011), suggesting the upper segments (proventriculus (Prov), gizzard (Giz) and possibly the crop) of the gastrointestinal tract (**GIT**) are the major active sites for phytase to have its’ effect. In addition, *in vitro* studies have demonstrated that phytate precipitates with Ca at pH higher than 4 (Jackman and Black, 1951; Nolan et al., 1987; Tamim et al., 2004), and decreases phytase dephosphorylation as a consequence. This highlights the importance of phytate degradation in the more acidic upper portions of the GIT (crop, Prov and Giz) in presence of phytase and the implications on total tract PP and total P digestion and absorption in the lower tract. Although several studies published recently examined the IP6 degradation in different GIT by accounting the feeding practice, ingredient specification, and phytase type factors (Svihus et al., 2010; Gao et al., 2013; Beeson et al., 2014; Walk et al., 2014; Zeller et al., 2015), dietary impacts of Ca, nonphytate P (**nPP**) and phytate concentration are rarely mentioned.

The objectives of the study were to determine the impact of dietary Ca, nonphytate P and PP concentrations at different phytase doses on 1) IP6 concentrations in crop, Prov and Giz, and ileum; and 2) the apparent IP6 disappearance in the distal ileum.

## MATERIALS AND METHODS

### Animals and Housing

All animal care procedures were approved by the University of Maryland Animal Care and Use Committee.

The experiment was conducted twice (block) in time with 3 replicates per treatment (**Trt**) represented in each block. For each block, day-old straight run Heritage 56M × fast-feathering Cobb 500F broiler chickens were obtained from a local commercial hatchery on the day of hatch and placed in floor pen rooms with artificial light and temperature control. A commercial type starter diet, formulated to contain 22.6% CP, 1.2% digestible Lys, 3010 kcal/kg ME, 1.0% Ca, and 0.48% nPP, that met or exceeded all NRC (1994) recommendations as well as average nutrient usage concentrations in the USA for 2012 (AgriStats 2012, end of year summary for 2012) was fed until d 10. On d 11, birds were individually weighed, grouped (10 birds/group) such that all groups had similar weight and within group chick weight variation was minimized, and placed into battery pens (Modified Petersime grower batteries, Petersime Incubator Co, Gettysburg OH) preassigned to Trt. The wire floored battery pens (width × depth × height; 99 cm × 68 cm × 37 cm) were equipped with nipple drinkers (2 per pen) and 2 external feed troughs (length × width × depth; 63.5 cm × 8.9 cm × 5.67 cm). On d 13, all birds in a pen were euthanized by cervical dislocation and contents from crop, Prov+Giz, and distal ileum were sampled, as described in the sampling section of the paper, from every bird.

Photoperiod was 24 h light (**L**):0 dark (**D**) from hatch to 3 d, 16L:8D from 4 to 7 d, 20L:4D from 8 to 11 d of age. Room temperature was kept at an average of 32°C from hatch to 3 d and brooder lamps were used to provide additional heat. Temperature was lowered by 1°C every 2 to 3 d such that bird comfort was maintained and temperature was 29°C at 11 d of age. Birds were checked twice daily and the weight of any dead bird, the remaining birds in the pen, and of the feed were recorded. Feed and water were offered for ad libitum consumption throughout the trial.

### Experimental Design and Diets

Two corn- and SBM-based mash basals with either low^1^ (geometric mean diameter (**d_gw_**) = 0.647 mm; standard deviation (**S_gw_**) = 0.719 mm) or high^2^ (**d_gw_** = 0.697; **S_gw_** = 0.731) in PP were formulated based on analyzed ingredient compositions (dry matter, ash, fat, amino acid, Ca, total P, and PP), mixed and analyzed for dry matter, macro minerals, protein, ether extract, and amino acids (Table 1). Particle size and distribution for diets and limestone were determined by ASAE method S319.3, 1997. Meat meal (5.07%) and rice bran (6.00%) were included in the low- and high-PP basals, respectively, to achieve the desired differences in PP concentration while maintaining similar concentrations of other nutrients. Based on analyzed Ca and P concentrations in the basal diets, pre-analyzed limestone^3^ (IMI Cal Pro, IN; **d_gw_** = 0.402 mm; **S_gw_** = 0.255 mm) and monocalcium phosphate^4^ (Kirby Agri, PA; d_gw_ = 0.759 mm; S_gw_ = 0.258 mm) were added to to achieve desired Ca and nPP concentrations in Trt diets. Final Trt diets contained 96.7% of either high or low PP basal. Titanium dioxide (TiO_2_) was added at 0.3% as the inert marker and Celite^®^ (World Minerals, CA) was used as a filler to achieve 100% for each Trt diet. The experiment was a 2 × 2 × 2 × 3 randomized block design with 2 Ca (0.7 and 1.0%), 2 PP (0.23 and 0.34%), 2 nPP (0.28 and 0.45%) and 3 phytase (0, 500 and 1,000 FTU/kg) concentrations resulting a total of 24 Trts (Table 2). Each Trt had 2 blocks with 3 replicate pens per block and 10 birds per pen). For each diet without phytase, a 6-phytase (from *Buttiauxella* sp.; Danisco Animal Nutrition, DuPont Industrial Biosciences, Marlborough, UK) was added on top, at 0, 500, or 1,000 FTU/kg to one of the three lots of the Trt and mixed so that the only difference among those lots was the phytase concentration. The starter and Trt diets were fed as mash throughout the trial.

**Table 1.** Ingredient and chemical composition of the basal diets.

	Basal (%, as-fed basis)	
Ingredient	Low PP	High PP
Corn	62.82	53.45
Soybean meal (48% CP)	28.5	35.10
Meat and bone meal	5.07	–
Rice bran	–	6.00
Soy oil	1.70	2.79
Salt	0.46	0.50
DL-Methionine, 99%	0.34	0.35
Biolys, 55%	0.57	0.44
L-Threonine, 98.5%	0.19	0.19
Choline chloride, 25%	0.19	0.18
Mineral Premix^1^	0.08	0.08
Vitamin Premix^2^	0.08	0.08
Limestone (36.5%)^3^	–	0.45
Monocalcium phosphate^4^	–	0.39
Total	100.00	100.00
Calculated (analyzed, mean±SD) concentrations (%)		
ME_n_, kcal/kg	3100	3100
Crude protein	22.5 (23.9 ± 0.8)	22.5 (25.9 ± 1.1)
Lysine	1.41 (1.43 ± 0.01)	1.41 (1.40 ± 0.04)
Methionine + Cystine	1.07 (1.06 ± 0.03)	1.05 (1.05 ± 0.03)
Calcium (Ca)	0.49 (0.50 ± 0.02)	0.49 (0.50 ± 0.02)
Total phosphorus (tP)	0.48 (0.52 ± 0.02)	0.59 (0.62 ± 0.01)
Inositol hexakisphosphate (IP6)	0.82 (0.89 ± 0.00)	1.22 (1.22 ± 0.00)
Phytate phosphorus (PP)	0.23 (0.25 ± 0.00)	0.34 (0.34 ± 0.00)
Non-phytate phosphorus (nPP)^5^	0.25 (0.27)	0.25 (0.28)

^1^Supplied per kg of diet: zinc from zinc sulfate, 85 mg; manganese from manganese sulfate, 107 mg; iron from iron sulfate, 21 mg; magnesium from magnesium oxide, 21 ppm; selenium from selenium sulfate, 0.32 ppm; copper from copper sulfate, 3 mg; iodine from calcium iodate, 4 mg.

^2^Supplied per kg of diet: vitamin A, 13,151 IU; vitamin D, 4,642 IU; vitamin E, 46.42 IU; vitamin B_12_, 0.02 mg; riboflavin, 15 mg; niacin, 62 mg; pantothenic acid, 21.7 mg; vitamin K_3_, 2.8 mg; folic acid, 1.87 mg; biotin, 0.13 mg; thiamine, 4 mg; pyridoxine, 5.4 mg.

^3^IMI Cal Pro, Irving Materias, IN. Analyzed Ca, 36.65%.

^4^Analyzed Ca and P: 16.06% and 21.95%, respectively.

^5^Concentration determined based on analyzed tP value minus analyzed PP.

**Table 2.** Formulated (calculated) and analyzed (determined) calcium (Ca), total phosphorus (P), phytate P (**PP**), nonphytate P (**nPP**) and phytase concentrations (mean±SD) in final diets (as is basis).^1^

Ca, %		P, %		PP^1^, %		nPP, %		Phytase, FTU/kg	
Fml^2^	Ana^2^	Fml	Ana	Fml	Ana	CAL^2^	Det^2^	Fml	Ana
0.7	0.70 ± 0.00	0.51	0.52 ± 0.00	0.23	0.24	0.28	0.28	0	<50
0.7	0.68 ± 0.00	0.62	0.63 ± 0.00	0.34	0.33	0.28	0.30	0	<50
0.7	0.70 ± 0.03	0.68	0.63 ± 0.02	0.23	0.24	0.45	0.39	0	<50
0.7	0.62 ± 0.01	0.79	0.79 ± 0.01	0.34	0.33	0.45	0.46	0	<50
1	0.89 ± 0.04	0.51	0.51 ± 0.01	0.23	0.24	0.28	0.27	0	<50
1	0.93 ± 0.02	0.62	0.63 ± 0.02	0.34	0.33	0.28	0.30	0	<50
1	0.89 ± 0.02	0.68	0.69 ± 0.01	0.23	0.24	0.45	0.45	0	<50
1	0.92 ± 0.06	0.79	0.78 ± 0.00	0.34	0.33	0.45	0.45	0	<50
0.7	0.70 ± 0.00	0.51	0.52 ± 0.00	0.23	0.24	0.28	0.28	500	511 ± 162
0.7	0.68 ± 0.00	0.62	0.63 ± 0.00	0.34	0.33	0.28	0.30	500	586 ± 62
0.7	0.70 ± 0.03	0.68	0.63 ± 0.02	0.23	0.24	0.45	0.39	500	471 ± 33
0.7	0.62 ± 0.01	0.79	0.79 ± 0.01	0.34	0.33	0.45	0.46	500	458 ± 12
1	0.89 ± 0.04	0.51	0.51 ± 0.01	0.23	0.24	0.28	0.27	500	545 ± 150
1	0.93 ± 0.02	0.62	0.63 ± 0.02	0.34	0.33	0.28	0.30	500	546 ± 18
1	0.89 ± 0.02	0.68	0.69 ± 0.01	0.23	0.24	0.45	0.45	500	562 ± 116
1	0.92 ± 0.06	0.79	0.78 ± 0.00	0.34	0.33	0.45	0.45	500	515 ± 106
0.7	0.70 ± 0.00	0.51	0.52 ± 0.00	0.23	0.24	0.28	0.28	1,000	989 ± 144
0.7	0.68 ± 0.00	0.62	0.63 ± 0.00	0.34	0.33	0.28	0.30	1,000	1,004 ± 52
0.7	0.70 ± 0.03	0.68	0.63 ± 0.02	0.23	0.24	0.45	0.39	1,000	945 ± 93
0.7	0.62 ± 0.01	0.79	0.79 ± 0.01	0.34	0.33	0.45	0.46	1,000	934 ± 151
1	0.89 ± 0.04	0.51	0.51 ± 0.01	0.23	0.24	0.28	0.27	1,000	1,010 ± 0
1	0.93 ± 0.02	0.62	0.63 ± 0.02	0.34	0.33	0.28	0.30	1,000	1,008 ± 159
1	0.89 ± 0.02	0.68	0.69 ± 0.01	0.23	0.24	0.45	0.45	1,000	936 ± 21
1	0.92 ± 0.06	0.79	0.78 ± 0.00	0.34	0.33	0.45	0.45	1,000	926 ± 45

^1^Calculated as analyzed percent PP in basal × 96.7% (inclusion of high or low PP basal in final diets). Analyzed PP concentrations in low and high PP basals are 0.25 ± 0.00 and 0.34 ± 0.00, respectively.

^2^Fml: formulated concentrations; Ana: analyzed concentrations; CAL: calculated concentrations calculated by the difference between Fml P and Fml PP; Det: determined concentrations.

### Sample Collection and Lab Analysis

At 13 d of age, all birds within a pen were sacrificed by cervical dislocation and all targeted GIT segment was removed within 30 s. Contents from crop, Prov+Giz, and ileum from each bird were taken into separated containers maintained in an ice bath. Until all birds in a pen were sampled, all containers were immediately frozen at –20°C to prevent any further effects of phytase on IP6. The last half of the ileum (distal half of the ileal segment encompassed between the Meckel's diverticulum and the ileocecal junction) was removed (Rodehutscord et al., 2012), placed on an ice cold marble slab and the contents gently expressed by flushing with ice cold distilled water. Digesta contents from different segment of GIT were pooled by pen by block, freeze-dried, ground (0.5-mm screen), and stored in air-tight containers at 4°C until analyzed.

Samples were analyzed in duplicate except where specified otherwise. Dry matter of diets and digesta contents were determined by drying overnight in a 100°C force-draft oven (Shreve et al., 2006). Dietary and ileal Ca and P were determined, in triplicate, after acid digestion and analyzed using inductively coupled plasma atomic emission spectrometry (ICP-AES; AOAC, 1999). Titanium (**Ti**) concentrations in diets and ileal digesta were determined by a colorimetric method adapted from Short et al. (1996) where samples were first ashed at 580°C and then digested in 7.4 M H_2_SO_4._ Crude protein and ether extract in basal diets were analyzed according to AOAC methods 990.03 (2003) and 920.39 (2003), respectively. Concentrations of amino acids were predicted by AMINONIR^®^ (Evonik Industries, Kennesaw, USA) in corn, SBM, meat meal and rice bran and analyzed by AMINOLab^®^ (Evonik Industries, Kennesaw, USA) in the basal diets. The IP6 concentrations in basal diets and digesta were analyzed according to the method described by Skoglund et al. (1997) and Skoglund et al. (1998). Phytase activities in all Trt diets were determined, in blind triplicate, according to the ISO 30024 (2009) procedure. One phytase unit (**FTU**) is the amount of enzyme that releases 1 μmol of inorganic orthophosphate from a sodium phytate substrate per minute at pH 5.5 and 37°C.

### Calculations

Apparent ileal IP6 disappearance was calculated based on the following formula using TiO_2_ as the inert marker:

\begin{eqnarray*}
{\rm IP}6\,{\rm disappearance} &=& \frac{{(IP6/TiO_2 )_d - (IP6/TiO_2 )_i }}{{(IP6/TiO_2 )_d }} \nonumber\\
&&\times 100\%
\end{eqnarray*}

Where (IP6/TiO_2_)_d_ is the ratio of IP6 to TiO_2_ in the diet and (IP6 / TiO_2_)_i_ is the ratio of IP6 to TiO_2_ in the ileal digesta.

Digested and remained IP6 was calculated as follows:

\begin{eqnarray*}
{\rm Digested}\,{\rm IP}6(\% ) &=& ({\rm IP}6\,{\rm disappearance}) \times {\rm Diet}\,{\rm IP}6 \\
{\rm Remained}\,{\rm IP}6(\% ) &=& {\rm Diet}\,{\rm IP}6 - {\rm Digested}\,{\rm IP}6
\end{eqnarray*}

where Diet IP6 is the analyzed IP6 concentration in either low- or high-PP basal × the basal inclusion in all diets (96.7%).

### Statistical Analysis

Data were analyzed by MIXED procedure of SAS (SAS Institute, 2008). Trt was considered as a fixed effect and block as a random effect. Effects of dietary Ca, nPP, PP, and phytase concentrations and their interactions were also determined. Tukey's (Tukey, 1949) adjustment was applied in all pair-wise comparisons to protect *P*-values. Pen within a block was the experimental unit and all calculations were generated based on pen averages. Significance was declared at *P* < 0.05.

## RESULTS

Analyzed concentrations of Ca, total P, phytic acid, and phytase were all close to formulated values, except analyzed Ca was lower (0.62%) in diets formulated with 0.7% Ca, 0.34% PP and 0.45% nPP, and determined nPP was lower (0.34%) in diets formulated with 0.7% Ca, 0.23% PP and 0.45% nPP than formulated Ca and calculated nPP concentrations, respectively (Table 2).

**Figure 1.**
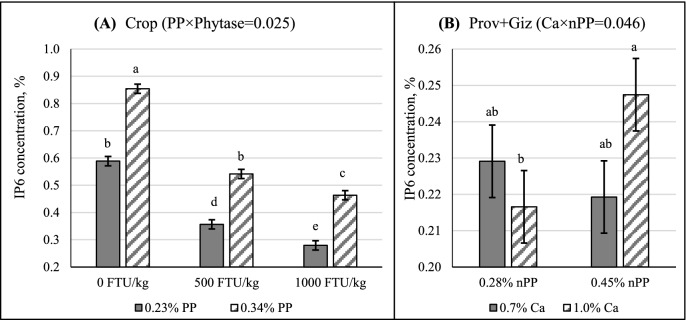
Impacts of interactions between (**A**) PP and phytase (crop); (**B**) Ca and nPP (proventriculus and gizzard; **Prov+Giz**); (**C**) nPP and phytase (ileum) and (**D**) Ca and PP (ileum) on inositol hexakisphosphate (**IP6**) concentrations in different segments of gastrointestinal tract (Mean±SEM). ^a–d^Means with different superscript letters differ. Tukey's test was used to separate group means within a treatment when the effect was significant.

Decreased IP6 concentrations in all 3 segments of GIT and improved ileal IP6 disappearance were seen with 500 FTU phytase/kg inclusion regardless of dietary Ca, nPP, or PP concentrations (Table 3; *P* < 0.05). Even though there was an interaction between PP and phytase on crop IP6 concentrations (Table 3; *P* < 0.05), responses to phytase inclusion were very similar between 0.23 and 0.34% PP (crop; Figure 1A). A Ca by nPP interaction was seen on Prov and Giz IP6 concentration (Figure 1B; *P* < 0.05). The IP6 concentration was higher as Ca concentration increased at 0.45% nPP, whereas there was no impact of Ca on IP6 concentration at 0.28% nPP. Across all nPP, PP and phytase concentrations, increasing Ca from 0.7 to 1.0% resulted in 7% increase in crop IP6 concentration (0.530 vs. 0.497%; *P* < 0.05). There was no impact of nPP on IP6 concentrations at any GIT segment, except in birds fed 1.0% Ca diets, Prov+Giz IP6 concentration was 14% higher at 0.45% than 0.28% nPP (*P* < 0.05). Despite the interactions between PP and phytase (crop; Table 3), and PP and Ca (ileum, Table 3), increasing dietary PP concentration from 0.23 to 0.34% increased IP6 concentration in all GIT segments (*P* < 0.05).

**Table 3.** Effects of dietary Ca, phytate P (**PP**), nonphytate P (**nPP**) and phytase concentrations on inositol hexakisphosphate (**IP6**) concentrations in different segments of gastrointestinal tract and ileal IP6 disappearance of birds fed experimental diets from 11 to 13 d of age.^1^

				IP6 concentration, %		
Ca^2^ %	PP^2^ %	nPP^2^ %	Phytase^2^ FTU/kg	Crop	P+G	Ileal IP6 disappearance^3,4^ %
0.70	0.23	0.28	0	0.584^b,c^	0.323^a–c^	18.4^e^
0.70	0.34	0.28	0	0.819^a^	0.361^a,b^	26.1^e^
0.70	0.23	0.45	0	0.577^b,c^	0.303^a–d^	32.0^e^
0.70	0.34	0.45	0	0.842^a^	0.386^a^	26.4^e^
1.00	0.23	0.28	0	0.565^b,c^	0.326^a–c^	16.7^e^
1.00	0.34	0.28	0	0.881^a^	0.369^a,b^	20.0^e^
1.00	0.23	0.45	0	0.630^b^	0.346^a,b^	30.7^e^
1.00	0.34	0.45	0	0.874^a^	0.369^a,b^	26.1^e^
0.70	0.23	0.28	500	0.345^f–h^	0.191^c–g^	78.0^a–c^
0.70	0.34	0.28	500	0.550^b–d^	0.250^b–f^	71.0^b–d^
0.70	0.23	0.45	500	0.371^e–g^	0.173^d–g^	76.6^a–c^
0.70	0.34	0.45	500	0.497^b–f^	0.196^c–g^	66.2^c,d^
1.00	0.23	0.28	500	0.330^f–h^	0.157^e,f^	78.8^a–c^
1.00	0.34	0.28	500	0.556^b–d^	0.168^e–g^	66.3^c,d^
1.00	0.23	0.45	500	0.379^d–h^	0.152^e–g^	67.9^c,d^
1.00	0.34	0.45	500	0.562^b,c^	0.267^a–e^	57.6^d^
0.70	0.23	0.28	1,000	0.206^h^	0.111^g^	91.5^a^
0.70	0.34	0.28	1,000	0.452^c–g^	0.132^f,g^	87.7^a^
0.70	0.23	0.45	1,000	0.286^g,h^	0.123^f,g^	91.4^a^
0.70	0.34	0.45	1,000	0.440^c–g^	0.135^f,g^	82.1^a–c^
1.00	0.23	0.28	1,000	0.303^g,h^	0.127^f,g^	90.4^a^
1.00	0.34	0.28	1,000	0.430^c–g^	0.153^e–g^	75.4^a–c^
1.00	0.23	0.45	1,000	0.321^f–h^	0.163^e–g^	84.1^a,b^
1.00	0.34	0.45	1,000	0.533^b–e^	0.185^c–g^	76.3^b^
**SEM**				0.0330	0.0240	3.20
***P*-values**				<0.001	<0.001	<0.001
**Main effect means^5^**						
Ca, %			0.7	0.497^b^	–	62.3^a^
			1.0	0.530^a^	–	57.5^b^
nPP, %			0.23	0.502	–	–
			0.34	0.526	–	–
PP, %			0.28	–	0.208^b^	–
			0.45	–	0.248^a^	–
Phytase, FTU/kg			0	–	0.348^c^	–
			500	–	0.194^b^	–
			1,000	–	0.141^a^	–
**Main effects and interaction *P*-values^6^**						
Ca				0.018	0.44	<0.001
nPP				0.08	0.30	0.86
PP				<0.001	0.001	<0.001
Phytase				<0.001	<0.001	<0.001
Ca×nPP				0.28	0.046	0.65
Ca×PP				0.66	0.99	0.22
Ca×Phytase				0.58	0.15	0.38
nPP×PP				0.30	0.54	0.17
nPP×Phytase				0.47	0.78	<0.001
PP×Phytase				0.025	0.39	0.002

^1^n = 6, Two blocks, 3 n/block, 10 birds/n.

^2^Formulated concentrations, analyzed concentrations are shown in Table 2.

^3^IP6 disappearance (%) = [(IP6/TiO_2_)_d_ – (IP6/TiO_2_)_i_]/(IP6/TiO_2_)_d_×100%. (IP6/TiO_2_)_d_ is the ratio of IP6 to TiO_2_ in the diet and (IP6/TiO_2_)_i_ is the ratio of IP6 to TiO_2_ in the ileal digesta.

^4^The amount of ileal IP6 disappeared can be calculated as: Disappeared IP6 (%) = IP6 disappearance×Diet IP6. Diet IP6 = Basal IP6 (0.89 or 1.22%) × 96.7% (basal inclusion rate).

^5^Main effects are only shown when there are no interactions.

^6^3 or 4-way interactions were not significant.

^a–h^Means within a column with different superscript letters differ (*P* < 0.05).

**Figure 2.**
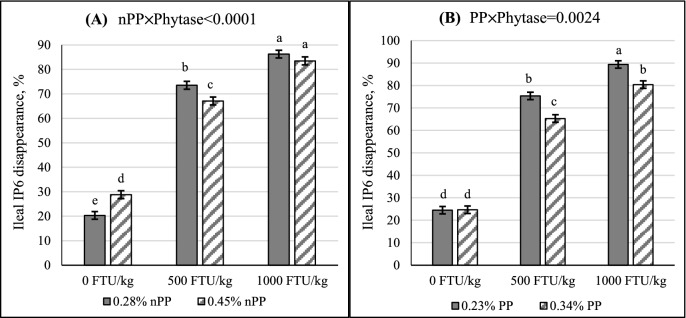
Impacts of interactions between (**A**) nPP and phytase and (**B**) PP and phytase on ileal inositol hexakisphosphate (**IP6**) disappearance (Mean±SEM). ^a-e^Means with different superscript letters differ. Tukey's test was used to separate group means within a treatment when the effect was significant.

Ileal IP6 disappearance was negatively affected by dietary Ca without interaction with other dietary factors, where it decreased from 62.3 to 57.5% when Ca increased from 0.7 to 1.0% (Table 3; *P* < 0.05). Impact of nPP concentration on IP6 disappearance was phytase dependent (Table 3; *P* < 0.05). Higher IP6 disappearance was seen with 0.45% nPP in the diets in the absence of phytase (Figure 2A; *P* < 0.05), whereas IP6 disappearance at 0.45% nPP was lower than 0.28% nPP with 500 FTU phytase/kg inclusion (*P* < 0.05). Increasing PP concentration reduced IP6 disappearance only when phytase, at either 500 or 1,000 FTU/kg, was added to the diets (Table 3; Figure 2B; *P* < 0.05). Similar to IP6 concentrations, adding phytase increased IP6 disappearance regardless of dietary Ca, PP, or nPP concentrations (Table 3; *P* < 0.05).

## DISCUSSION

In order to correctly calculate digestibility/retention using marker method, a marker shall flow at an identical rate as the testing nutrients, remain undigested through the gut, be non-toxic, and be fully recovered in the digesta (Kotb and Luckey, 1972). Passage rate in the upper GIT depends, however, largely on the particle size, meaning that large particles tend to be retained in the Giz until they are broken down to a certain size (Clemens et al., 1975; Hetland et al., 2002). It is questionable whether or not the flow of powder-like markers that tend to be water mixable, such as Ti and Cr, can be representative of the total diet in the upper tract due to the large differences in particle size and solubilities or mixabilities (Clemens et al., 1975; Svihus et al., 2002; Zeller et al., 2015). Therefore, digesta from crop and Prov+Giz was only analyzed for IP6 concentration while disappearance defined as change in concentration of IP6 in the segment digesta vs. diet was only determined in the ileum in the current study.

### IP6 Concentration in the Upper GIT

Despite the amount of work has been done to determine phytase efficacies in terms of P and PP utilization in ileum, information on the IP6 degradation in the upper GIT is much less. A recent study from Walk et al. (2014) showed that IP6 concentration in Giz was 98% lower in birds fed 500 U *E. coli* phytase/kg (0.04 μmol/g) compared to birds fed the same diet (0.82% Ca and 0.30% available P) without phytase inclusion (1.91 μmol/g) at 21 d of age (fed from hatch to 21 d). Similar degree of reductions were also reported by Beeson et al. (2014) that with 1995 FTU *E. coli* phytase/kg inclusion, Giz IP6 concentration decreased by more than 90% compared to those fed the same Ca (1.28%) and nPP (0.42%) corn-SBM diets without phytase (fed from hatch to 21 d of age). Substantial decline in IP6 concentration in Prov+Giz was also observed the current study, where IP6 concentration was, on average, 60% lower with 1,000 FTU/kg inclusion compared to birds fed non-phytase diet. The different degree of IP6 reduction seen between the current study as compared to Walk et al. (2014) and Beeson et al. (2014) may relate to 1) lower Giz IP6 concentration in non-phytase Trt in Walk et al. (2014) study (0.126%) as compared to the average of 0.325% in the 0.23% PP Trts in the current study, in spite of a higher dietary PP concentration was suggested based on ingredient composition (61.31% corn, 31.91% SBM, and 2% dried distillers grains with solubles); and 2) possible higher dose used (1995 FTU phytase/kg) in Beeson et al. (2014), even though the phytases’ origin (*E. coli* vs. *Buttiauxella*) may also account for the differences. In addition, because commercial phytases maintain high activities between pH 2 and 5 (Morales et al., 2011; Yu et al., 2004), time of sampling and the rapidity of terminating phytase activity after sampling may also have substantial impacts on IP6 concentration in the upper tract.

With the average pH of 5.5 in crop and 3 in Prov and Giz (Ravindran, 2013), these segments are expected to be the major sites for microbial phytase digestion in poultry species (Liebert et al., 1993; Yu et al., 2004). In laying hens, the highest phytase activity for two commercial phytases was found in crop, followed by Prov and Giz (Gao et al., 2013). This results support findings from the current study, where 38 and 49% reduction in crop IP6 concentration was found as a result of 500 and 1,000 FTU phytase/kg inclusion, respectively. Similarly, a significant decrease in crop IP6 concentration overtime (0 to 180 min after feeding) was also reported in broilers fed pelleted diet with 1,000 FTU *E. coli* phytase/kg inclusion (Svihus et al., 2010).

Several in vitro studies have clearly showed that phytate maintains relatively high solubility at pH lower than 4, irrespective of Ca concentration, but the solubility decreases substantially when pH is above 4, which is also negatively affected by Ca concentration (Grynspan and Cheryan, 1983; Nolan et al., 1987; Grynspan and Cheryan, 1989). The interaction between Ca and phytate demonstrated *in vitro* can possibly explain the different Ca effect seen in crop and Prov+Giz, where increased Ca led to higher IP6 concentration in crop but had no impact on that in Prov+Giz. Interestingly, increasing dietary nPP from 0.28% to 0.45% was found to increase IP6 concentration in Prov+Giz at 1.0% Ca (Figure 1B). Because this interaction between Ca and nPP was not dependent on PP or phytase, it is unlikely the increased IP6 concentration was a result of product inhibition from higher dietary nPP. Instead, it is likely because of elevated pH due to increased Ca concentration (Amerah et al., 2014), which, in term decreased IP6 solubility and/or promoted Ca-PP chelation (Grynspan and Cheryan, 1983; Nolan et al., 1987; Tamim et al., 2004). The potential exists for the soluble IP6 to move faster than the non-soluble and large particles of the diet. This, though, would occur in the presence or absence of phytase, as IP6 needs to be in solution for phytase to act on.

### IP6 Disappearance in the Distal Ileum

It is evident that as digesta moved to the lower GIT, there was an accumulation of undigested IP6 in the distal ileum irrespective of dietary factors. The average IP6 disappearance in the absence of phytase was 24.55% with no difference seen among Trts. The results suggested that, in the absence of phytase, diet compositions had no impact on IP6 disappearance, which contradicts results reported by others. In general, decreased PP disappearance is found with the increase of Ca. For example, Tamim et al. (2004) reported a 63% decreased in PP disappearance from 69.2 to 25.4% when dietary Ca increased from 0.18 to 0.65% (0.31% PP). Another study from Amerah et al. (2014) found a similar negative impact of Ca on PP disappearance between birds fed 0.51 and 0.68% Ca when diets containing 0.19% nPP and 0.32% PP were fed to broiler birds (5 to 21 d of age). However, the Ca effect seen from the above studies should be interpreted carefully, as the Ca concentration from neither of the studies was higher than the low Ca concentration used in the current study (0.70% Ca). In the Amerah et al. (2014) study, no Ca effect on PP disappearance was found when Ca was above 0.68% which is in agreement with results seen in the current study. Kim et al. (2013) reported that there was no further negative impact on P digestibility when dietary Ca was above 0.78%, suggesting that a saturation of the chelation between Ca and phytate occur and that this saturation is related to phytate concentration in the diet. Therefore, the lack of Ca effect on IP6 hydrolysis in the ileum may be because all potential chelation sites in the IP6 present were already taken up by Ca even at 0.70% Ca.

Phytase addition substantially improved IP6 disappearance regardless of Ca, nPP or PP concentrations. With added 1,000 FTU phytase/kg to 0.23% PP diets, 89% IP6 disappeared in the distal ileum, while this value reduced to 80% when PP increased to 0.34%. In contrast to our observations, Cowieson et al. (2006) reported greater IP6 hydrolysis in birds fed higher IP6 diets both in the presence and absence of phytase. However, in their study, the IP6 disappearance was determined by modified precision feeding, where a phytic acid solution (50%) was added to a suspension of 5 g casein in 50 mL of distilled water while the phytic acid or phytate in the current study originated from the feed ingredients. It very likely that the conflicting results seen between Cowieson et al. (2006) and the current study are related to the nature of phytic acid and feeding method (Ravindran et al., 1995; Rutherfurd et al., 2004).

In addition, IP6 disappearance was 9% lower when dietary nPP increased from 0.28 to 0.45% with 500 FTU phytase/kg inclusion, but this reduction in IP6 disappearance was not seen when phytase dose was increased to 1,000 FTU/kg. Recently, Zeller et al. (2015) also reported a similar interaction between added nPP and phytase dose, where adding nPP from MCP (0.09% nPP) in the presence of 500 FTU of an *E. coli* phytase/kg resulted in a lesser degree of IP6 degradation in the ileum but the added nPP had no impact on IP6 degradation when phytase dose increased to 12500 FTU/kg. Results from the current work and that of Zeller et al. (2015) support the concept that the impact of nPP on phytate degradation is phytase dose dependent.

## CONCLUSION

Results from the current study demonstrated that phytase addition improved IP6 degradation in all segments of gastrointestinal tract examined in this trial with crop, Prov and Giz being the most active sites for phytase hydrolysis. Because IP6 has the highest potential to chelate minerals and protein as compared to lower IP esters (Persson et al., 1998), effectiveness of phytase to remove the first phosphate groups from phytate would have significant implications on mineral and protein digestibilities. Our results showed that the effectiveness of phytase to degrade IP6 was significantly affected by Ca, nPP and PP concentrations in the diet. To minimize the anti-nutrient properties of phytate and maximize phytase efficacy, phytase dose may need to be adjusted accordingly depending on dietary Ca, nPP and PP concentrations.

## Footnotes

1 Particle size distribution of low PP basal: >2.360 mm, 1.50%; 2.000–2.360 mm, 3.45%; 1.700–2.000 mm, 5.65%; 1.400–1.700 mm, 8.85%;1.180–1.400 mm; 8.65%; 1.000–1.180 mm, 9.65%; 0.850–1.000 mm, 9.65%; 0.710–0.850 mm, 7.40%; 0.500–0.710 mm, 17.25%; 0.300–0.500 mm, 14.20%; 0.250–0.300 mm, 0.65%; 0.075–0.250 mm, 6.50%; <0.075 mm, 6.60%.

2 Particle size distribution of high PP basal: >2.360 mm, 1.75%; 2.000–2.360 mm, 3.60%; 1.700–2.000 mm, 6.10%; 1.400–1.700 mm, 9.35%;1.180–1.400 mm; 9.10%; 1.000–1.180 mm, 9.70%; 0.850–1.000 mm, 10.00%; 0.710–0.850 mm, 8.75%; 0.500–0.710 mm, 17.40%; 0.300–0.500 mm, 13.10%; 0.250–0.300 mm, 0.55%; 0.075–0.250 mm, 4.75%; <0.075 mm, 5.85%.

3 Particle size distribution of limestone used in the basal and Trt diets: >1.180 mm, 0.07%; 1.000–1.180 mm, 0.71%; 0.850–1.000 mm, 5.36%; 0.710–0.850 mm, 9.74%; 0.600–0.710 mm, 10.07%; 0.500–0.600 mm, 15.96%; 0.425–0.500 mm, 10.84%; 0.355–0.425 mm, 13.10%; 0.300–0.355 mm, 9.36%; 0.250–0.300 mm, 7.88%; 0.150–0.250 mm, 10.20%; 0.075–0.150 mm, 4.99%; <0.075 mm, 1.72%.

4 Particle size distribution of monocalcium used in the basal and Trt diets: >1.180 mm, 0.82%; 1.000–1.180 mm, 8.64%; 0.850–1.000 mm, 34.83%; 0.710–0.850 mm, 24.04%; 0.600–0.710 mm, 17.22%; 0.500–0.600 mm, 9.13%; 0.300–0.500 mm, 3.81%; <0.300 mm, 1.51%.
